# Developing search strategies for clinical practice guidelines in SUMSearch and Google Scholar and assessing their retrieval performance

**DOI:** 10.1186/1471-2288-7-28

**Published:** 2007-06-30

**Authors:** Andrea Haase, Markus Follmann, Guido Skipka, Hanna Kirchner

**Affiliations:** 1 Institute for Quality and Efficiency in Health Care (Institut für Qualität und Wirtschaftlichkeit im Gesundheitswesen [IQWiG]), Dillenburger Str. 27, 51105 Cologne, Germany

## Abstract

**Background:**

Information overload, increasing time constraints, and inappropriate search strategies complicate the detection of clinical practice guidelines (CPGs). The aim of this study was to provide clinicians with recommendations for search strategies to efficiently identify relevant CPGs in SUMSearch and Google Scholar.

**Methods:**

We compared the retrieval efficiency (retrieval performance) of search strategies to identify CPGs in SUMSearch and Google Scholar. For this purpose, a two-term GLAD (GuideLine And Disease) strategy was developed, combining a defined CPG term with a specific disease term (MeSH term). We used three different CPG terms and nine MeSH terms for nine selected diseases to identify the most efficient GLAD strategy for each search engine. The retrievals for the nine diseases were pooled. To compare GLAD strategies, we used a manual review of all retrievals as a reference standard. The CPGs detected had to fulfil predefined criteria, e.g., the inclusion of therapeutic recommendations. Retrieval performance was evaluated by calculating so-called diagnostic parameters (sensitivity, specificity, and "Number Needed to Read" [NNR]) for search strategies.

**Results:**

The search yielded a total of 2830 retrievals; 987 (34.9%) in Google Scholar and 1843 (65.1%) in SUMSearch. Altogether, we found 119 unique and relevant guidelines for nine diseases (reference standard). Overall, the GLAD strategies showed a better retrieval performance in SUMSearch than in Google Scholar. The performance pattern between search engines was similar: search strategies including the term "guideline" yielded the highest sensitivity (SUMSearch: 81.5%; Google Scholar: 31.9%), and search strategies including the term "practice guideline" yielded the highest specificity (SUMSearch: 89.5%; Google Scholar: 95.7%), and the lowest NNR (SUMSearch: 7.0; Google Scholar: 9.3).

**Conclusion:**

SUMSearch is a useful tool to swiftly gain an overview of available CPGs. Its retrieval performance is superior to that of Google Scholar, where a search is more time consuming, as substantially more retrievals have to be reviewed to detect one relevant CPG. In both search engines, the CPG term "guideline" should be used to obtain a comprehensive overview of CPGs, and the term "practice guideline" should be used if a less time consuming approach for the detection of CPGs is desired.

## Background

An exploding quantity of information, increasing time constraints, and the inadequacy of traditional sources of information underline the importance for clinicians to search efficiently for evidence-based and up-to-date medical information to support diagnostic, prognostic, and therapeutic decision-making processes [1]. Clinical practice guidelines (CPGs) are "systematically developed statements to assist practitioner and patient decisions about appropriate health care for specific clinical circumstances" [2], and are becoming an increasingly familiar part of medical practice [3]. Factors that make searches for CPGs problematic include incomplete indexing in bibliographic databases, as well as the difficulties clinicians encounter in selecting optimal search strategies [4,5]. CPG-specific databases exist, such as the NGC (National Guideline Clearinghouse), which includes CPGs that meet defined inclusion criteria and have been published within the previous 5 years [6]. However, so far no search engine is available that searches all guideline databases (e.g. NGC, NHS Clinical Knowledge Summaries [7], Canadian Medical Association Infobase – Clinical Practice Guidelines [8]).

The free-access Internet search engines SUMSearch and Google Scholar are widely used when searching for medical information. SUMSearch was developed by the University of Texas in 1999 [9,10]. It searches the Internet for evidence-based medical information, scanning databases (MEDLINE, DARE, and NGC) as well as various high-impact medical journals [11]. SUMSearch provides validated integrated search filters such as the diagnosis filter developed by Haynes et al [12]. To automate searching, SUMSearch combines meta- and contingency searching. Meta-searching is designed to scan multiple databases and sites simultaneously, and returns one single retrieval document to the user. If too many retrievals are obtained, more restrictive searches (contingency searches) are conducted by activating additional filters. Conversely, if the number of retrievals is too small, SUMSearch adds more databases to the search [13]. The retrievals in SUMSearch are presented in a box with categories arranged from narrative reviews with a broad perspective to publications that are more specific and more difficult to read [11,14]. Within the categories, the search results are organised according to database, and are ranked predominantly by publication date in descending order.

Google Scholar is a search engine that was launched in November 2004 by Google Inc. It is available in the beta test-version, which is in continuous transition [15]. Google Scholar is organised according to a so-called federated search [16]: its web crawlers search, process, and index information in the World Wide Web, incorporating it into a single repository, and it refers to this repository to process a search. Google Scholar was developed to provide "a simple way to broadly search for scholarly literature" across many sources (e.g. peer-reviewed papers, books, academic publishers, and universities) [17]. However, further details are not provided; for example, the sources or the search algorithms have not been disclosed, and the term "scholarly" has not been defined [18-22]. In contrast to SUMSearch, Google Scholar presents the search results in a ranked list, the retrievals sorted according to relevance, taking the number of citations into account [17]. However, this system is not strictly applied and may be biased by the high number of citations of older records [19,23]. In both Google Scholar and SUMSearch, the search strategy and search results cannot be saved.

Our analysis was motivated by the fact that we had previously not identified studies that compared search strategies for CPGs in SUMSearch and Google Scholar by means of diagnostic parameters. The model for our study was provided by the analysis methods introduced by the Hedges group to detect different types of studies in different databases [12,24-31]. The aim of this study was to provide clinicians with useful search strategies to identify CPGs in SUMSearch and Google Scholar.

## Methods

We compared the retrieval performance (efficient detection of relevant CPGs) of a two-term search strategy in SUMSearch and Google Scholar, using a manual review of retrievals as a reference standard. Our research focussed on nine different diseases currently being evaluated by the German health authorities with regard to their suitability for inclusion in disease management programmes (obesity, osteoporosis, rheumatoid arthritis, Parkinson disease, multiple sclerosis, alcoholism, depressive disorder, schizophrenia, and attention deficit disorder). A "relevant" CPG had to fulfil predefined criteria (e.g. the inclusion of diagnostic or therapeutic recommendations). "Efficient" meant detecting as many unique and relevant CPGs as possible in a given period of time (which varies depending on the time available to the user).

The two primary performance parameters of the evaluation were the completeness of the detected pool of CPGs (measured with the parameter "sensitivity"), and the number of retrievals that had to be read (number needed to read; NNR) to find one relevant CPG. These two measures of retrieval performance represent the (possibly contradictory) situation of a clinician searching for CPGs: i) he or she has sufficient time for a complete review of retrievals to preferably detect all available relevant CPGs on a topic; ii) he or she would like to detect CPGs on a specific condition at short notice while treating a patient. In the former case, the completeness of the search result is decisive; in the latter, the time invested is. Specificity (the ability of a strategy to disregard non-relevant retrievals) was defined as a secondary performance parameter.

**Figure 1:**
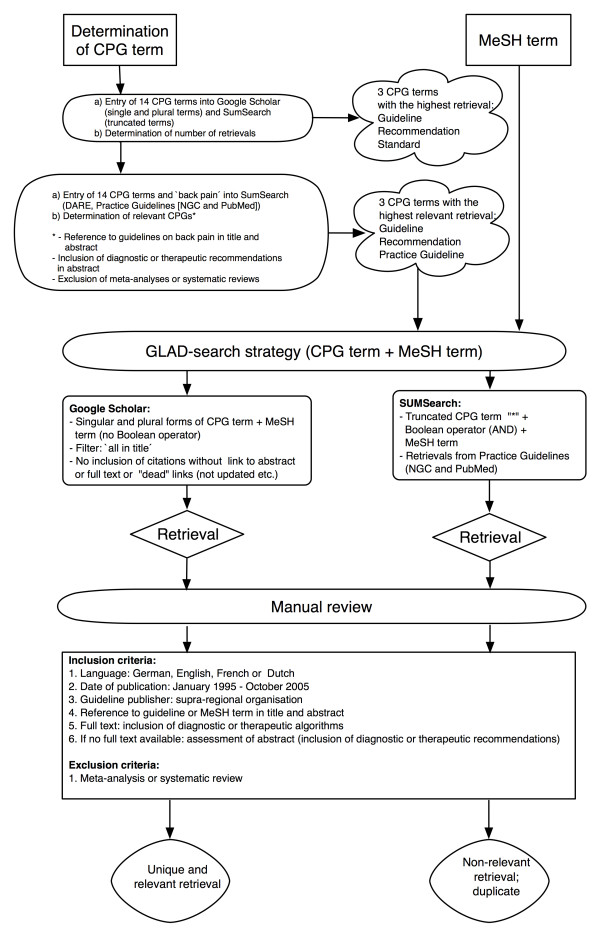
Flowchart of study methodology.

The individual methodological procedures of the evaluation were continuously developed during the study, according to the experience gained while working with the search engines. These procedures were not specified a priori and were therefore conducted as an iterative process. An overview of the study methodology is shown in Figure 1. We used a two-step approach: (1) the development of a GLAD search strategy (preliminary study); (2) the application of this strategy and the comparison of retrieval performance (main study). The study was conducted in October 2005.

### Preliminary study

#### Developing the search strategies

First, we entered 14 terms commonly used in guideline searches [32] into SUMSearch and Google Scholar (Table 1). The three terms that produced the most retrievals in both search engines were "guideline", "practice guideline" and "standard" (**Retrievals SUMSearch, Retrievals Google Scholar **in Table 1). We then checked these 14 terms for relevance in combination with the MeSH term "back pain" in SUMSearch (DARE, NGC, and PubMed), using the test term "back pain" as an example of a common disorder and a substitute for the specific disease terms. We restricted this analysis to SUMSearch, since Google Scholar does not solely provide retrievals of medical relevance. We classified a retrieval as "relevant" if the title and abstract of the retrieval included a reference to a CPG on back pain, and the abstract included diagnostic or therapeutic recommendations. Publications identified as meta-analyses or systematic reviews were excluded. The inclusion of a CPG was independent of the methodology of the guideline development (e.g. CPGs based on systematic reviews were not excluded). We recorded the number of retrievals for each term. The terms "guideline", "practice guideline", and "recommendation" produced the highest number of relevant retrievals (**CPG term + "back pain" **in Table 1), and were therefore defined as the three CPG terms to be used as the first term of the GLAD search strategy. The second term was one of the nine disease-specific MeSH terms mentioned earlier.

**Table 1:** Determination of clinical practice guideline terms for the GLAD search strategy in the preliminary study

**14 commonly used search terms^†^**	**Retrievals SUMSearch^‡^**	**Retrievals Google Scholar^‡ ^**(estimated number of retrievals)^§^	**Relevant retrievals CPG term + "back pain"^|| ^**(guidelines in the top 20 [DARE 24] retrievals)
Guideline/-s/-*	340,105	1,954,000	58
Practice guideline/-s/-*	139,385	984,000	46
Recommendation/-s/-*	162,239	1,892,000	35
Standard/-s/-*	1,384,650	18,560,000	30
Clinical pathway	3,332	420,000	0
Clinical protocol	76,530	572,000	0
Clinical standard	64,009	872,000	0
Clinical recommendation	3,649	64,900	5
Consensus	62,113	970,000	7
Clinical consensus	12,401	263,000	0
Consensus (SUMSearch: AND) development conferences	6,189	49,700	6
Position paper	7,115	1,740,000	0
Clinical (SUMSearch: AND) position paper	1,085	272,000	0
Good (SUMSearch: AND) clinical practice	6,487	768,000	0

^† ^The terms 'guideline', 'practice guideline', 'recommendation' and 'standard' were entered into SUMSearch and Google Scholar with the truncation '*', and as singular and plural terms.

^‡ ^The number of retrievals produced by the respective CPG terms per search engine in the preliminary study.

^§ ^Defined by Google Scholar as "Results 1–10 of about...."

^|| ^The number of guidelines identified in SUMSearch by the combination of a CPG term and the MeSH term "back pain".

* = truncation; CPG = Clinical Practice Guideline; GLAD = GuideLine And Disease.

### Main study

#### Application of the search strategies

The developers of SUMSearch assessed predictors of successful searches for medical evidence and showed that searches were twice as likely to succeed when no more than two terms were included or MeSH terms were used [11]. On the basis of their specifications for an effective search in a search engine, we developed a two-term search strategy combining one of three different CPG terms and one of nine MeSH terms for a specific disease. This resulted in 27 search combinations for SUMSearch and Google Scholar. The components of the search strategy – CPG term and MeSH term – prompted us to call this approach the GLAD (GuideLine And Disease) strategy.

We adapted the syntax of the GLAD strategy to the individual requirements of each search engine (Figure 1). In SUMSearch, the truncated CPG terms were combined with the Boolean operator "AND" (1. guideline*, 2. recommendation*, 3. practice guideline*; AND MeSH term). The search was restricted to the category "Practice Guidelines" (NGC and PubMed). Since the PubMed practice guidelines section of SUMSearch does not provide citations, we used the link provided by SUMSearch to search for guidelines at PubMed. DARE had initially been included in the preliminary study, but as no guidelines were retrieved (only systematic reviews and meta-analyses), it was subsequently excluded from the main study.

In Google Scholar, the single or plural CPG terms were used without an operator (1. guideline/s; 2. recommendation/s; 3. practice guideline/s; MeSH term). We used the advanced interface and selected "in the title". We entered the combination of each CPG term (single or plural) and each MeSH term into the search box "with all of the words". We used the "all in title" restriction, as prior informal searching in Google Scholar had produced an excessive amount of retrievals without this limit.

The retrievals for the nine diseases were pooled. The application of the GLAD search strategy therefore resulted in three retrieval pools (one for each CPG term applied) per search engine.

#### Defining the reference standard

After collecting the raw retrievals in SUMSearch and Google Scholar, we defined a reference standard by manually reviewing all retrievals (links) from the retrieval list of each search engine. Each raw retrieval was regarded as a reading unit that had to be reviewed to identify unique and relevant CPGs. Since the GLAD strategies were to be tested against a reference standard, the retrievals had to be unique (i.e., excluding duplicates).

The unique and relevant CPGs of both search engines formed the reference against which each of the three CPG-term search strategies applied in SUMSearch and Google Scholar was tested. As duplicates also involved a relevant input of work and time for the reviewer (each one had to be assessed individually), they were also considered in the analysis (Table 2).

**Table 2:** Allocation of retrievals in the manual review (3 GLAD-strategies, 9 diseases)

**Allocation of retrievals**	**Unique and relevant retrievals**	**Non-relevant retrievals and duplicates**	**Total**
**SUMSearch**			

**Strategy tested**	unique and relevant retrievals; no duplicates	non-relevant retrievals removed from manual review; duplicates of: - relevant retrievals - non-relevant retrievals	raw retrievals; duplicates between PubMed and NGC
**Strategies not tested**	unique and relevant retrievals; no duplicates	non-relevant retrievals removed from manual review; duplicates of and between: - relevant retrievals - non-relevant retrievals	raw retrievals; duplicates of and between retrievals
**All strategies**	unique and relevant retrievals; no duplicates	non-relevant retrievals removed from manual review; duplicates of and between: - relevant retrievals - non-relevant retrievals	raw retrievals; duplicates of and between retrievals

**Google Scholar**			

**Strategy tested**	unique and relevant retrievals; no duplicates	non-relevant retrievals removed from manual review; duplicates of: - relevant retrievals - non-relevant retrievals	raw retrievals; duplicates between singular and plural
**Strategies not tested**	unique and relevant retrievals; no duplicates	non-relevant retrievals removed from manual review; duplicates of and between: - relevant retrievals - non-relevant retrievals	raw retrievals; duplicates of and between retrievals
**All strategies**	unique and relevant retrievals; no duplicates	non-relevant retrievals removed from manual review; duplicates of and between: - relevant retrievals - non-relevant retrievals	raw retrievals; duplicates of and between retrievals

The full-text articles of the retrievals were screened and assessed; if these were not available, abstracts were reviewed. Retrievals were declared "relevant" if the detected CPG fulfilled specific criteria with regard to content, language, and publication date (Figure 1). One reviewer classified the retrievals according to these criteria; these results were perused by three additional reviewers. The retrievals from Google Scholar and SUMSearch were then finally classified into two categories: either "unique and relevant" or "non-relevant or duplicate".

**Figure 2:**
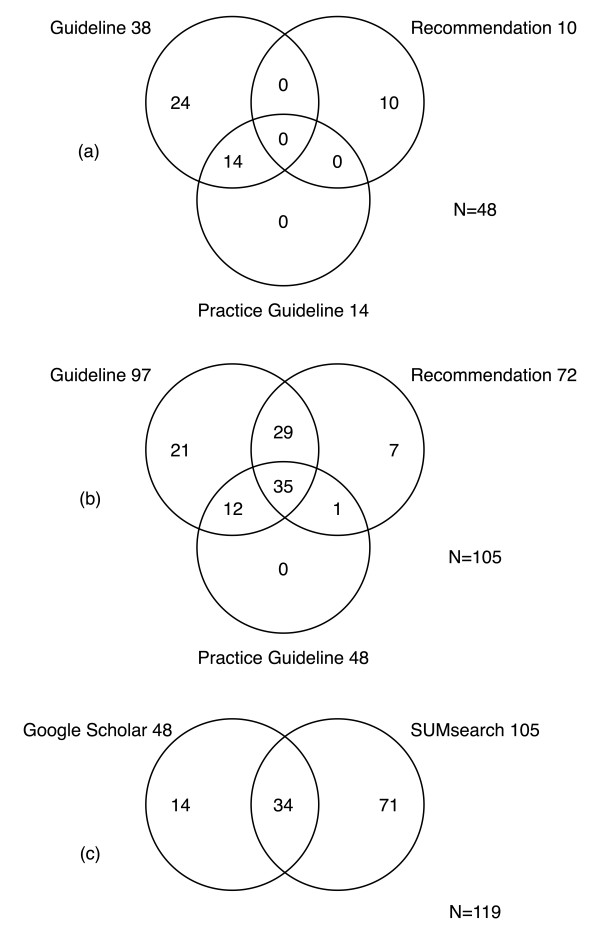
**Intersections of unique and relevant clinical practice guidelines of nine tested diseases**. (a) Per CPG term in Google Scholar **(b) **Per CPG term in SUMSearch **(c) **All CPG terms combined (SUMSearch and Google Scholar). CPG = clinical practice guideline.

We recorded the number of identified CPGs per GLAD strategy (for all diseases) and per search engine, and documented the corresponding intersections of CPGs (Figure 2).

#### Diagnostic test parameters

The sensitivity, specificity, and NNR of the three GLAD strategies were calculated per search strategy in each search engine following the terminology of diagnostic tests, using the defined gold standard as a reference (Table 3) [26]. It should be noted that these diagnostic parameters will change depending on the reference pool.

**Table 3:** Formula for calculating retrieval performance parameters for search strategies*

	**Manual review**	
**Search terms**	Meets criteria (unique, relevant CPGs)	Does not meet criteria (non-relevant CPGs or duplicates)
Detected	a	b
Not detected	c	d

*Following the methodology of the Hedges group (see text): sensitivity = a/(a+c); specificity = d/(b+d); precision = a/(a+b); total sample (all unscreened reviewed retrievals) = a+b+c+d; NNR = 1/precision.

CPG = clinical practice guideline.

Sensitivity for a given strategy is the proportion of identified unique and relevant guidelines among the total number of unique and relevant retrievals identified by the reference search; specificity is the proportion of non-relevant retrievals and duplicates not retrieved by this strategy among the total number of non-relevant retrievals and duplicates not retrieved by the reference search; precision is the proportion of retrieved guidelines that are unique and relevant among the total number of retrievals. The NNR (1/precision) is defined as the number of non-relevant and duplicate retrievals that have to be screened to find one relevant retrieval [33]. For all parameters, 95% confidence intervals were calculated based on simple normal approximation for proportions.

## Results

The application of the three GLAD search strategies per search engine for each of the nine diseases (totalling 27 searches per engine) yielded a total of 2830 retrievals (for the nine pooled diseases); 987 (34.9%) in Google Scholar and 1843 (65.1%) in SUMSearch. The manual review identified a total of 119 unique and relevant CPGs (reference standard) in both search engines (Table 4). SUMSearch detected 105, and Google Scholar detected 48 relevant guidelines, of which 71 and 14 were unique and relevant, respectively (Figure 2).

**Table 4:** Retrievals obtained by application of GLAD search strategies in SUMSearch and Google Scholar

**Strategy Search terms**	**Meets criteria**	**Does not meet criteria**	**Total**
**SUMSearch**			1843^†^

Guideline*			
Detected	97	697	794
Not detected	22	2014	2036
Recommendation*			
Detected	72	643	715
Not detected	47	2068	2115
Practice guideline*			
Detected	48	286	334
Not detected	71	2425	2496

**Google Scholar**			987^†^

**Guideline/s**			
Detected	38	595	633
Not detected	81	2116	2197
**Recommendation/s**			
Detected	10	214	224
Not detected	109	2497	2606
**Practice guideline/s**			
Detected	14	116	130
Not detected	105	2595	2700

Total	119	2711	2830

^† ^Number of pooled retrievals for the nine MeSH terms in each search engine.

* = truncation; GLAD = Guideline And Disease.

Table 5 shows the retrieval performances of the three GLAD strategies in SUMSearch and Google Scholar. In SUMSearch, the search strategy including the term "guideline" yielded the highest sensitivity (81.5%), the lowest specificity (74.3%), and an NNR of 8.2 (meaning that about 8 retrievals had to be reviewed to find one unique and relevant guideline). The search strategy including the term "practice guideline" yielded the highest specificity (89.5%), the lowest sensitivity (40.3%), and the lowest NNR (7.0). The search strategy including the term "recommendation" did not achieve the best result for any performance parameter – neither primary nor secondary – and yielded a sensitivity of 60.5%, a specificity of 76.3%, and a NNR of 9.9.

**Table 5:** Retrieval performance of search strategies in SUMSearch and Google Scholar^†^

**Search strategy**	**Sensitivity (%)**	**Specificity (%)**	**NNR^‡^**
**SUMSearch**			
Guideline*	81.51 (74.53 to 88.49)	74.29 (72.64 to 75.94)	8.18 (6.90 to 10.05)
Recommendation*	60.50 (51.72 to 69.28)	76.28 (74.67 to 77.89)	9.93 (8.14 to 12.72)
Practice guideline*	40.34 (31.52 to 49.16)	89.45 (88.29 to 90.61)	6.96 (5.52 to 9.43)
**Google Scholar**			
Guideline/s	31.93 (23.56 to 40.30)	78.05 (76.50 to 79.60)	16.67 (12.76 to 24.04)
Recommendation/s	8.40 (3.42 to 13.38)	92.11 (91.09 to 93.13)	22.42 (13.97 to 56.82)
Practice guideline/s	11.76 (5.98 to 17.54)	95.72 (94.96 to 96.48)	9.29 (6.21 to 18.38)

^† ^95% confidence intervals in brackets.

^‡ ^NNR = number needed to read.

* = truncation.

In Google Scholar, the search strategy including the term "guideline" yielded the highest sensitivity (31.9%), the lowest specificity (78.1%) and a NNR of 16.7. The search strategy including the term "practice guideline" yielded the highest specificity (95.7%), a sensitivity of 11.8%, and the lowest NNR (9.3). The search strategy including the term "recommendation" yielded the lowest sensitivity (8.4%), a specificity of 92.1%, and the highest NNR (22.4). In the two latter strategies, the low sensitivity seems to be a trade-off for the high specificity.

In summary, the GLAD search strategies showed a better retrieval performance in SUMSearch than in Google Scholar. The performance pattern between search engines was similar: in both search engines, the best results for the primary performance parameters were achieved by strategies including the term "guideline" (highest sensitivity) and "practice guideline" (lowest NNR). This means that the former strategy had the most comprehensive detection of unique and relevant CPGs, i.e. it detected the highest number of unique and relevant retrievals among the reference retrievals. The latter required the lowest time investment in a search, i.e. it required the lowest number of retrievals to identify a relevant retrieval. None of the tested strategies combined the highest sensitivity and lowest NNR. The best results for the secondary performance parameter (highest specificity) were also achieved by the strategy including the term "practice guideline", meaning that this strategy disregarded the highest proportion of non-relevant retrievals.

With regard to the NNR, about 30% more time was required in Google Scholar than in SUMSearch to detect a relevant CPG (using the strategy including "practice guideline") (Table 5). Concerning the primary performance parameter "sensitivity" and the search term "guideline", Google Scholar only detected about 40% of the relevant CPGs that SUMSearch identified (Tables 4 and 5).

Google Scholar showed better results only for the secondary performance parameter "specificity" (identification of about 7% more non-relevant retrievals with the search strategy including the term "practice guideline"; Tables 4 and 5).

## Discussion

The aim of this study was to provide clinicians with useful search strategies to efficiently identify relevant CPGs in SUMSearch and Google Scholar. We therefore compared the retrieval performance of GLAD (GuideLine And Disease) strategies in SUMSearch and Google Scholar, using a manual review of retrievals as a reference standard. As the reference standard was solely defined by means of the manual search of the raw retrievals in SUMSearch and Google Scholar and only includes CPGs identified by these engines, it cannot be universally transferred to searches in other search engines or bibliographic databases. It should also be noted that since conducting our search, changes have been made to both SUMSearch and Google Scholar. Therefore, the replicability of results may be affected. A further limitation of this study may be due to the fact that the reviewers were not blinded as to which search engine produced the CPG. They were not blinded because, as stated, the methodological procedures were not specified a priori and were conducted as an iterative process. However, as none of the reviewers previously routinely used either SUMSearch or Google Scholar, we believe it is unlikely that bias caused by a preference for a specific search engine was a relevant factor.

### Search methods in SUMSearch and Google Scholar

The GLAD strategies showed a better retrieval performance when applied in SUMSearch than in Google Scholar. However, one needs to consider that our approach was based on the specifications developed for searches in SUMSearch; therefore, the transfer of a "SUMSearch-specific" strategy to Google Scholar might have been unfavourable for its retrieval performance. To our knowledge, there is no 'best search strategy' for CPGs – or one that is better than our GLAD strategy – available for Google Scholar.

The strength of SUMSearch lies in its expertise in the medical sciences. Since its launch, the search strategies of users have been continuously reviewed. This process has led to the current recommendations for search strategies. SUMSearch's structural characteristic as a meta-search engine in scientific sources supports the specificity of its retrieval performance. The handling of retrievals in SUMSearch is convenient. The links presented in the search results are in reference format and lead directly to the article or abstract of the retrieval.

It remains open as to whether the ongoing changes, such as the inclusion of additional filters, in the structure of Google Scholar (which is still in its beta version) will lead to an improvement in retrieval performance. Due to its intuitive approach, Google Scholar has been described as especially quick in locating frequently cited articles and the proverbial "needle in a haystack" [18]. Despite this commendation, search strategies for CPGs in Google Scholar need to be improved and defined more specifically. The handling of retrievals can be quite laborious: Web links refer to on- and offline sources, as well as to open-access sources and sources where registration is required (e.g. publishers' websites) [22], and sometimes lead to "dead links". Abstracts are not always accessible and open-access items are not specially marked. Furthermore, Google Scholar's coverage is incomplete; less than 10% of PubMed records are searched, and it only partially covers publishers' and societies' archives – again, the character of the archives is not disclosed in detail [19,20,22,23]. Changes in PubMed, which plans to make its web page titles more descriptive [34], may improve Google Scholar's performance in this database. Google Scholar also searches the contents of databases with a time-lag. It has been noted in online panels that the time-lag for indexing PubMed has been reduced from a year to about five months [35,36].

### Retrieval performance results

In SUMSearch as well as in Google Scholar, we found that the term "practice guideline" is the CPG-term that should be included in a GLAD strategy by the clinician under time pressure who is looking for quick answers to a clinical problem by means of a CPG. Although this strategy may not identify all relevant CPGs, it has a high probability of detecting a sufficient number to answer the question posed. This is because it has a low NNR and a high specificity, and is therefore likely to detect relevant retrievals quickly without having to review an excessive number of non-relevant ones. If the clinician wants to gain a comprehensive overview of CPGs on a certain topic, a GLAD search strategy including the term "guideline" should be used; this was the term with the highest sensitivity. Although this strategy is more time consuming, it considerably increases the chances of detecting a large number of relevant CPGs. However, the dilemma in which the searcher finds him- or herself, time pressure on the one hand and the desire to detect as many relevant CPGs as possible on the other hand, cannot be solved to complete satisfaction.

## Conclusion

Google Scholar is not a useful tool to search efficiently for CPGs. The advantage of being intuitive does not make up for the laborious and time consuming handling of the retrievals when screening for CPGs. We recommend SUMSearch as a starting point for a search to gain an overview of available CPGs. It specialises in the medical field and has the advantages of a meta-search engine that searches medical databases simultaneously. The retrievals are accessed quickly and reliably by links, enabling swift screening. However, neither SUMSearch nor Google Scholar can replace the commonly used CPG searches in portals/websites of organisations that publish CPGs and bibliographical databases.

The developed GLAD strategy is easy to use and to remember. It is applicable in both SUMSearch and Google Scholar. In SUMSearch, however, the GLAD strategy shows a superior retrieval performance.

We did not identify the "ideal" CPG term for a GLAD strategy. The term "guideline" should be used if the aim is to detect a comprehensive pool of CPGs, and "practice guideline" should be used if the aim is to rapidly identify CPGs.

In future, the introduction of a standardised index term for CPGs, analogous to MeSH terms, may facilitate identification by search engines and help to improve retrieval performance. A further vision for the combination of the advantages of both search principles – the federated search of Google Scholar and the meta-search of SUMSearch – could lead to the development of a 'Medical Internet Portal'. In combination with a standardised CPG-index term, relevant CPGs could be identified more quickly and comprehensively.

## List of abbreviations

CPG: Clinical Practice Guideline

DARE: Database of Abstracts of Reviews of Effects

GLAD: GuideLine And Disease

MeSH: Medical Subject Heading

NGC: National Guideline Clearinghouse

NNR: Number Needed to Read

## Competing interests

The author(s) declare that they have no competing interests.

## Authors' contributions

This study was initiated by HK and MF. All authors provided intellectual content to the literature search and the data analysis. The literature search was conducted by AH. Data analysis was conducted by AH and GS. The draft of the manuscript was prepared by AH. All authors commented on and approved the final manuscript.

## Pre-publication history

The pre-publication history for this paper can be accessed here:


